# Risk perception of severity or death from COVID-19: a systematic review of the factors associated

**DOI:** 10.3389/fpubh.2025.1543629

**Published:** 2025-05-26

**Authors:** Rander Junior Rosa, Rubia Laine de Paula Andrade, Letícia Perticarrara Ferezin, Mônica Chiodi Toscano de Campos, Heriederson Sávio Dias Moura, Thais Zamboni Berra, Natacha Martins Ribeiro, Titilade Kehinde Ayandeyi Teibo, André Luiz Teixeira Vinci, Felipe Mendes Delpino, Miguel Ángel Fuentealba Torres, Ricardo Alexandre Arcêncio

**Affiliations:** ^1^University of São Paulo at Ribeirão Preto College of Nursing, Ribeirão Preto, São Paulo, Brazil; ^2^University of Brasília, Brasília, Brazil; ^3^Federal University of Pelotas, Capão do Leão, Rio Grande do Sul, Brazil; ^4^Faculty of Nursing and Obstetrics of the Universidad de los Andes, Santiago, Chile

**Keywords:** COVID-19, severe illness, associated factors, perception, risk

## Abstract

**Background:**

Health risk perception and factors associated with the severity or death from COVID-19 were key elements that influenced individuals' protective behaviors during the pandemic. Understanding these perceptions is crucial for public health guidelines that encourage preventive measures and improve an outbreak response strategy. Thus, this systematic review aimed to identify factors associated with the perception of risk of severity or death from COVID-19.

**Methods:**

A systematic review was conducted with an article search performed in March 2024 across five databases, utilizing both controlled and free vocabulary. Studies published from 2020 onward were included. Two reviewers independently selected articles, with disagreements resolved by a third reviewer. The data were extracted using a structured form, and the findings were synthesized narratively. The studies included in the review underwent a methodological quality assessment using tools proposed by the Joanna Briggs Institute.

**Results:**

Nineteen articles were included in the review. Among the factors most frequently associated with the perception of severe illness or death from COVID-19 were advanced age, female gender, personal experience or witnessing of adverse COVID-19 outcomes, the presence of chronic non-communicable diseases, and lower educational attainment.

**Conclusion:**

The study highlights that the perception of risk for COVID-19 severity or death varied according to age, gender, and prior experiences with the disease. Such findings can guide healthcare practices and contribute to the formulation of public policies, strengthening responses to future public health crises.

**Systematic review registration:**

identifier CRD42024444734, https://www.crd.york.ac.uk/PROSPERO/view/CRD42024444734.

## 1 Introduction

COVID-19, caused by the novel Severe Acute Respiratory Syndrome Coronavirus 2 (SARS-CoV-2), poses a significant challenge to global public health. Common symptoms of the disease include dry cough, fatigue, sore throat, and fever, which can progress to severe clinical complications, such as pneumonia, requiring supportive care measures like mechanical ventilation (1).

The risk perception is related to severe complications or death from COVID-19, which refers to an individual's perceived vulnerability to the severity of the disease (2) may influence the adoption of protective and/or risky behaviors, along with fear, prosocial attitudes, the perceived effectiveness of preventive measures, and trust in healthcare professionals and science (3). Risk perception may therefore continue to influence people's daily decisions, potentially reducing their adherence to mask-wearing, social distancing, and vaccination. Moreover, as health threats become more imminent and severe, risk perceptions also increase, significantly impacting mental health, with individuals, in the case of COVID-19, tending to report elevated levels of worry and fear (4, 5).

Risk perception is subjective and varies among individuals. It can be heightened or reduced based on several factors, including the manner in which information about COVID-19 is disseminated by media outlets and health authorities, personal experiences with the disease and observed instances of COVID-19 progression in others (6). The concept of risk extends beyond cultural worldviews and cognitive processes (7–10), being influenced by factors such as trust in governmental institutions, scientific bodies, and medical professionals, as well as an individual's understanding of government strategies and their sense of personal and collective efficacy (3, 11).

Trust in scientific and public health institutions can influence risk perception and adherence to preventive measures in various ways, depending on how this trust is measured and contextualized (8). An individual's risk perception and subsequent behavioral responses during the pandemic are further shaped by the interplay of interpersonal trust, media communication, and personal experiences (7). Understanding these factors is crucial for developing effective public health interventions and improving communication strategies. This knowledge can help increase adherence to protective measures and, consequently, prevent the spread of communicable diseases such as COVID-19.

Despite the extensive literature on COVID-19 (3, 7, 8, 10–13), there remains a gap in the systematic synthesis of data on risk perception and its determinants across diverse populations. Moreover, current research often provides fragmented insights or focuses on single-population studies, lacking a comprehensive approach to comparing findings across various contexts and demographic profiles.

A systematic review could address this gap by consolidating evidence on variations in risk perception, identifying common trends, and highlighting how these perceptions influenced adherence to preventive measures. Additionally, by synthesizing existing data, this review may offer a clearer understanding of the psychological, social, and cultural factors that have impacted public response to health crises, thereby informing future pandemic preparedness strategies.

Given the scarcity of a comprehensive study on the associate factors influencing risk perception of COVID-19 severity or mortality, particularly in the context of a global health crisis, this study aimed to analyze, through a systematic literature review, the factors that shape the perception of risk regarding COVID-19 severity or mortality, with a focus on the interplay between sociocultural and behavioral factors.

## 2 Methods

This review was conducted following the guidelines of the Preferred Reporting Items for Systematic Reviews and Meta-Analysis (PRISMA) (14) and the steps outlined in the “Methodological Guidelines: Development of Systematic Review and Meta-Analysis of Comparative Observational Studies on Risk Factors and Prognosis” (Brazil, 2014). The study protocol was registered with the International Prospective Register for Systematic Reviews (PROSPERO ID: CRD42024444734).

### 2.1 Research question

The investigation was guided by the “PEO” strategy, which poses the question: “What are the factors associated with the perception of risk of severity or death from COVID-19?” In this context, “PEO” is an abbreviation that breaks down as follows: “P” for Population (adult individuals), “E” for Exposure (associated factors), and “O” for Outcome (risk perception regarding the development of severe illness or death from COVID-19). The details of this approach are presented in Table 1, showing the structure of the PEO.

**Table 1:** PEO framework for research question.

**Description**	**Abbreviation**	**Components**
Population	P	Adult/Older Adult individuals
Exhibition	E	Associated factors
Outcome	O	Perception of risk of severity or death from COVID-19

Source: Prepared by the author.

### 2.2 Eligibility criteria

Studies published in all languages between 2020 and 2024 were included, focusing on research involving humans aged 18 years or older. Only studies that aimed to identify and analyze factors associated with the perception of risk regarding severity or death from COVID-19 were considered, regardless of methodological quality. The review included analytical observational studies, irrespective of the country of origin and/or publication.

The studies excluded from the review were those that: (1) did not provide information on the outcome of interest (risk perception of severe illness or death from COVID-19); (2) assessed and provided information solely on factors associated with COVID-19 infection; and (3) offered information on factors associated with COVID-19 protective measures.

### 2.3 Sources of information

In March 2024, the bibliographic survey was carried out in the following electronic databases: LILACS (Latin American and Caribbean Literature in Health Sciences); MEDLINE (Medical Literature Analysis and Retrieval System online) via PubMed (Public/Publisher MEDLINE); Embase (Excerpta Medica Database); Web of Science (Web of Science Core Collection) via CAPES journal portal; and Scopus (SciVerse Scopus).

### 2.4 Search strategy

The keywords and descriptors for the searches were extracted from the Health Sciences Descriptors (DeCS) and Medical Subject Headings (MeSH) and through previous searches in the databases. The search strategies were individually adjusted for each database, using a combination of Boolean operators (“AND” and “OR”), as shown in Table 2.

**Table 2:** Search strategies used in the systematic review of factors associated with the perception of risk of severity or death from COVID-19, according to the databases consulted, Brazil, 2024.

**Database**	**Search strategies**
LILACS	(“new coronavirus” OR “covid 19” OR “covid-19” OR “wuhan coronavirus” OR coronavirus OR “sars-cov-2” OR “sars cov-2” OR “sars cov 2” OR “sars-cov 2” OR “novo coronavírus” OR coronavirus OR “nuevo coronavirus”) AND (“risk perception” OR “risk perceptions” OR “perception of risk” OR “perceived risk” OR “perceived risks” OR “perceived severity” OR “perception of health risk” OR “percepção de risco” OR “percepções de risco” OR “risco percebido” OR “riscos percebidos” OR “gravidade percebida” OR “percepción de riesgo” OR “percepciones de riesgo” OR “riesgo percibido” OR “riesgos percibidos” OR “gravedad percibida”) AND (“severity of illness index” OR “disease severity” OR “severe course” OR “severe disease” OR death OR fatality OR dying OR mortality OR “fatal disease” OR “índice de gravidade da doença” OR “gravidade da doença” OR “curso grave” OR “doença grave” OR morte OR mortes OR óbito OR óbitos OR fatalidade OR mortalidade ou “doença fatal” OR “índice de gravedad de la enfermedad” OR “gravedad de la enfermedad” OR “evolución grave” OR “enfermedad grave” OR muerte OR muertes OR letalidad OR mortalidad OR “enfermedad mortal”) AND (db:(“LILACS”)) AND (year_cluster:[2020 TO 2024])
MEDLINE	((“new coronavirus”[All Fields] OR “covid-19”[All Fields] OR “covid-19”[All Fields] OR “wuhan coronavirus”[All Fields] OR (“coronavirus”[MeSH Terms] OR “coronavirus”[All Fields] OR “coronaviruses”[All Fields]) OR “sars cov 2”[All Fields] OR “sars cov 2”[All Fields] OR “sars cov 2”[All Fields] OR “sars cov 2”[All Fields]) AND (“risk perception”[All Fields] OR “risk perceptions”[All Fields] OR “perception of risk”[All Fields] OR “perceived risk”[All Fields] OR “perceived risks”[All Fields] OR “perceived severity”[All Fields] OR “perception of health risk”[All Fields]) AND (“severity of illness index”[All Fields] OR “disease severity”[All Fields] OR “severe course”[All Fields] OR “severe disease”[All Fields] OR “death”[All Fields] OR (“fatal”[All Fields] OR “fatalities”[All Fields] OR “fatality”[All Fields] OR “fatally”[All Fields]) OR “dying”[All Fields] OR (“mortality”[MeSH Terms] OR “mortality”[All Fields] OR “mortalities”[All Fields] OR “mortality”[MeSH Subheading]) OR “fatal disease”[All Fields])) AND (2020:2024[pdat])
Web of science	“new coronavirus” OR “covid 19” OR “covid-19” OR “wuhan coronavirus” OR coronavirus OR “sars-cov-2” OR “sars cov-2” OR “sars cov 2” OR “sars-cov 2” (Topic) and “risk perception” OR “risk perceptions” OR “perception of risk” OR “perceived risk” OR “perceived risks” OR “perceived severity” OR “perception of health risk” (Topic) and “severity of illness index” OR “disease severity” OR “severe course” OR “severe disease” OR “death” OR fatality OR dying OR mortality OR “fatal disease” (Topic) and 2024 or 2023 or 2022 or 2021 or 2020 or 2019 (Publication Years)
Scopus	TITLE-ABS-KEY (“new coronavirus” OR “covid 19” OR “covid-19” OR “wuhan coronavirus” OR coronavirus OR “sars-cov-2” OR “sars cov-2” OR “sars cov 2” OR “sars-cov 2”) AND TITLE-ABS-KEY (“risk perception” OR “risk perceptions” OR “perception of risk” OR “perceived risk” OR “perceived risks” OR “perceived severity” OR “perception of health risk”) AND TITLE-ABS-KEY (“severity of illness index” OR “disease severity” OR “severe course” OR “severe disease” OR “death” OR fatality OR dying OR mortality OR “fatal disease”) AND PUBYEAR > 2019 AND PUBYEAR <2025
Embase	#1 ‘new coronavirus‘ OR ‘covid 19′/exp OR ‘covid 19′ OR ‘covid-19'/exp OR ‘covid-19' OR ‘wuhan coronavirus‘/exp OR ‘wuhan coronavirus‘ OR ‘coronavirus‘/exp OR coronavirus OR ‘sars-cov-2'/exp OR ‘sars-cov-2' OR ‘sars cov-2'/exp OR ‘sars cov-2' OR ‘sars cov 2'/exp OR ‘sars cov 2' OR ‘sars-cov 2'/exp OR ‘sars-cov 2'
	#2 ‘risk perception'/exp OR ‘risk perception' OR ‘risk perceptions' OR ‘perception of risk' OR ‘perceived risk'/exp OR ‘perceived risk' OR ‘perceived risks' ‘perceived severity'/exp OR ‘perceived severity' OR ‘perception of health risk'
	#3 ‘severity of illness index'/exp OR ‘severity of illness index' OR ‘disease severity'/exp OR ‘disease severity' OR ‘severe course' OR ‘severe disease' OR ‘death'/exp OR ‘death' OR ‘fatality'/exp OR fatality OR ‘dying'/exp OR dying OR ‘mortality'/exp OR mortality ‘fatal disease'/exp OR ‘fatal disease'
	#4 #1 AND #2 AND #3
	#5 #4 AND (2020:py OR 2021:py OR 2022:py OR 2023:py OR 2024:py) AND [embase]/lim

### 2.5 Screening process

The selection process utilized Rayyan QCRI software, chosen for its intuitive interface and collaborative screening tools. As a first step, duplicate publications were eliminated. Two independent reviewers (RJR and LPF) conducted the selection based on pre-defined criteria, where they considered research objectives, study type and population characteristics. Studies were included if they met the following criteria: original research, published in English, Portuguese, or Spanish and involved human samples, used quantitative or qualitative methodologies. Studies were excluded if they were review articles, based on non-representative samples and failed to meet methodological standards. The screening process occurred in two stages: reading titles and abstracts and, full analysis of selected texts. In cases of disagreement, a third reviewer (RLPA) was consulted to ensure selection consistency. This process ensured high agreement between reviewers and maintained the methodological robustness of the review.

### 2.6 Data extraction process

The data extraction process was carried out meticulously by two reviewers (RJR and LPF), who utilized a standardized form developed by the research team. This form included the following items: authors, year of publication, country of the study, study objective, study type, study location, population characteristics, data analysis, and study results. The reviewers independently extracted data from all included studies, and the collected data were compared, with a third reviewer involved in case of any disagreements. The obtained data were then organized and systematically entered into a table in Word software, allowing for a clear and structured visualization of the results.

### 2.7 Assessment of the quality of studies

The studies were evaluated for methodological quality by the researcher (RJR) using the Joanna Briggs Institute (JBI) critical appraisal tool for cross-sectional studies (15). This tool consists of eight questions addressing inclusion criteria, participant and setting descriptions, exposure measurement, standardized criteria, confounding factors, strategies for confounding factors, outcome measurement, and statistical analysis. Each study's score was evaluated as: “yes,” if the criterion was clearly met; “no,” if the criterion was not met; “unclear,” if it was not clear whether the criterion was met; and “not applicable.” A “yes” response was scored as “1,” while the responses “no,” “unclear,” and “not applicable” were scored as “0.” The quality score for each study was calculated and expressed as a percentage (16).

### 2.8 Summary of results

The results of the studies were narratively synthesized.

## 3 Results

**Figure 1:**
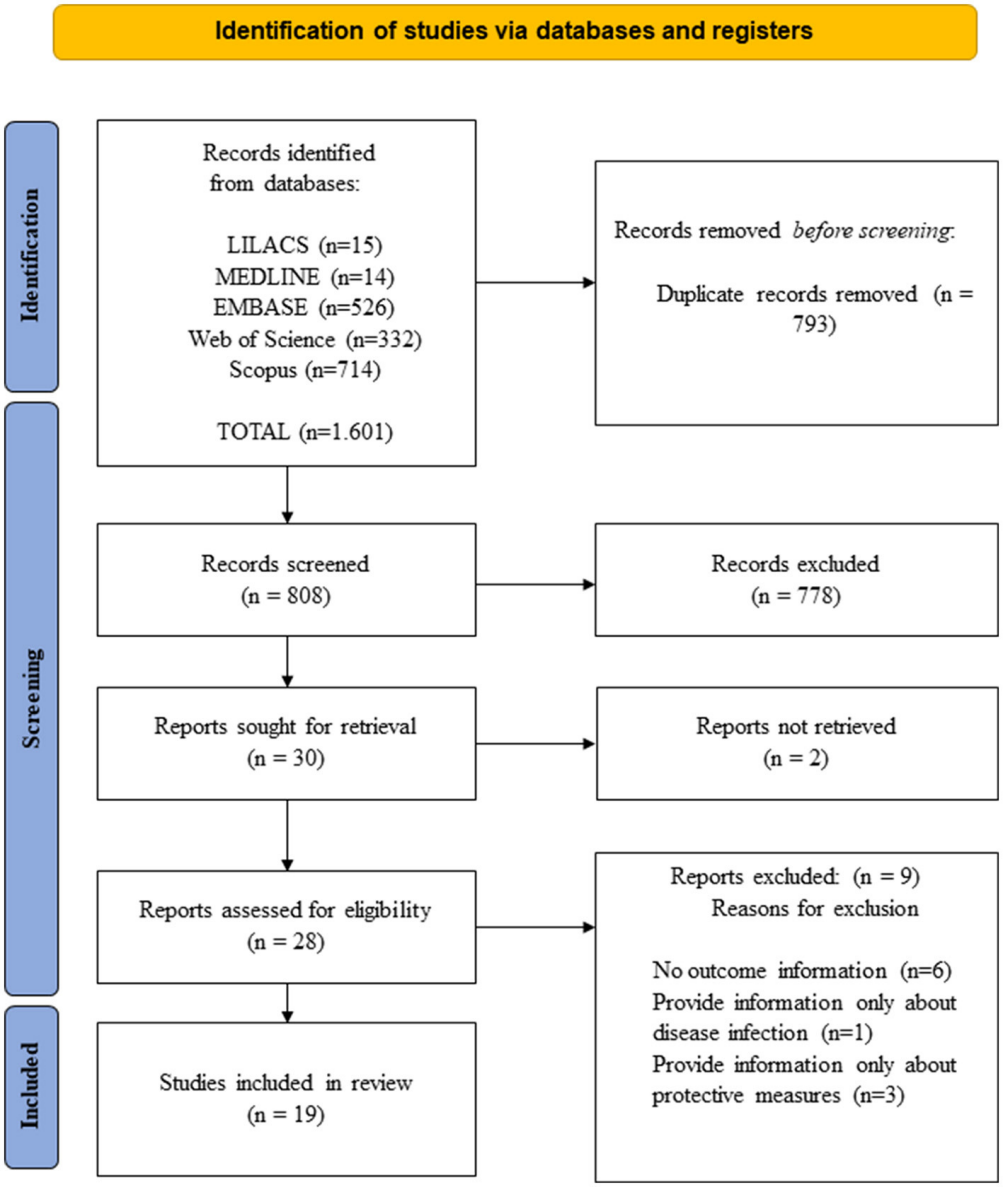
PRISMA flowchart of study selection in the systematic review on factors associated with the perception of risk regarding severity or death from COVID-19, Brazil, 2024. Source: Adapted from Page et al. (14).

Through the search conducted in the databases, 1,601 publications were identified. After the removal of duplicates, 808 studies were screened for relevance regarding inclusion in the study. A total of 778 studies were excluded after reading the titles, two studies were excluded because they could not be located in full-text, and nine studies were excluded after full-text reading. Thus, 19 studies that addressed factors associated with the perception of risk regarding severity or death from COVID-19 were included in the review. Figure 1 illustrates the steps taken during the study selection process.

Two studies on the topic were published in 2020 (17, 18), eight in 2021 (19–25, 45), four in, (26–29) and five in 2023 [(30–34); Table 2].

**Figure 2:**
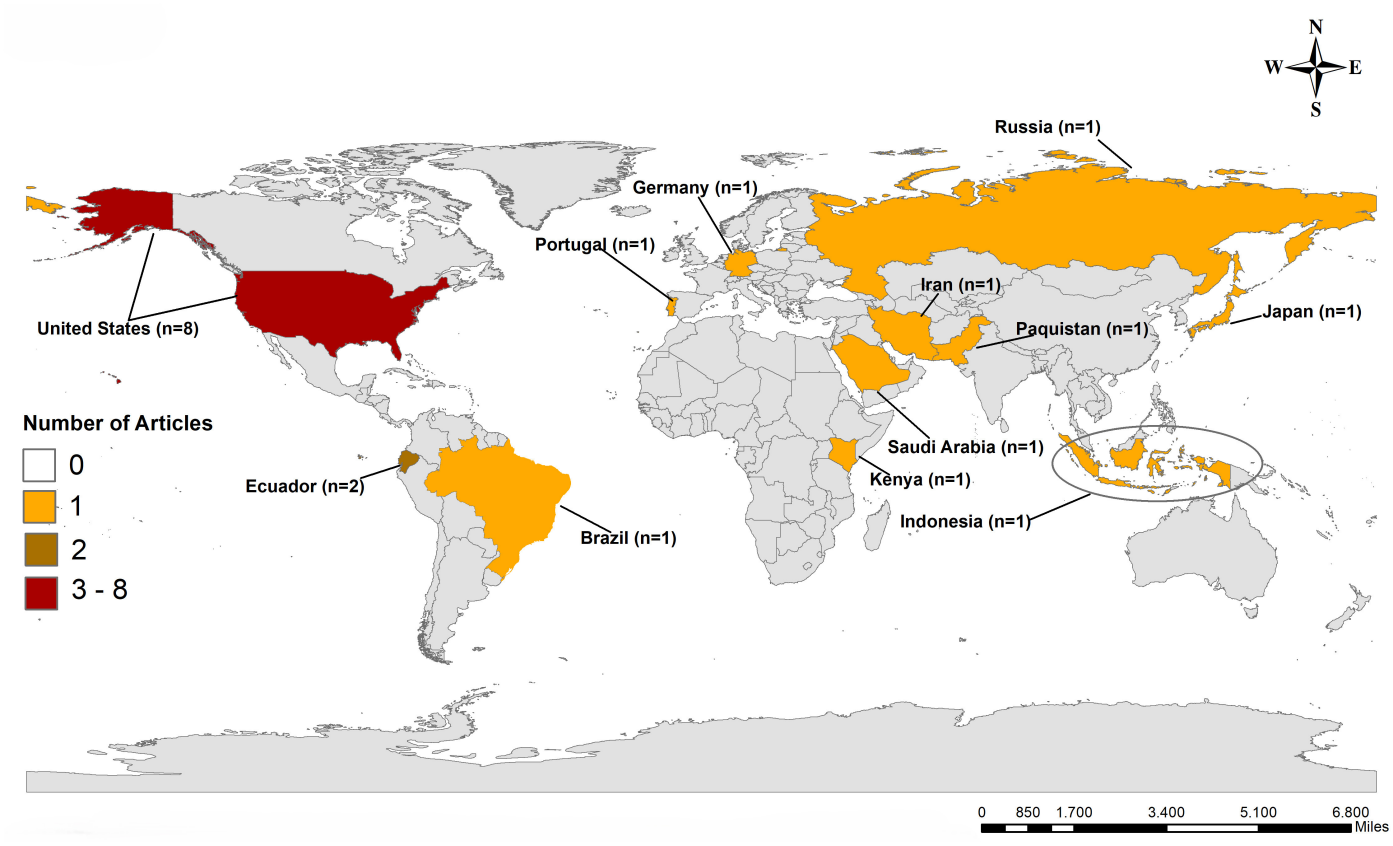
Geographical distribution of studies included in the systematic review on factors associated with perceived risk of severity or death from COVID-19, according to databases consulted, Brazil, 2024.

Eight studies included in the review were conducted in the United States (17, 18, 20, 22, 26, 31, 34, 45), two in Ecuador (23, 32), and one each in Brazil (27), Germany (25), Portugal (24), Pakistan (19), Russia (33), Japan (29), Saudi Arabia (30), Iran (21), Kenya (32) and Indonesia (28) (Table 2, Figure 2).

All studies included in the systematic review were cross-sectional in design, with the study populations and main results presented in Table 3.

**Table 3:** Characteristics and main results found in published studies on factors associated with the perception of risk of severity or death from COVID-19, according to the consulted databases, Brazil, 2024.

**Reference**	**Periodical**	**Objective of the study**	**Study design**	**Population/Country of study**	**Main results**
De Bruin and Bennett (17)	American Journal of Preventive Medicine	To examine perceived risks for COVID-19 infection and mortality and whether these perceptions were associated with protective behaviors	Cross-sectional study	6.684/Estados Unidos	Perceived risk of death from COVID-19 was higher in people aged ≥65 years (*p < * 0.001), females (*p < * 0.01), black people (African American; *p < * 0.01), people with low/middle income (*p < * 0.001) and in people without higher education (*p < * 0.001)
Shauly et al. (18)	Journal of Medical Internet Research	Assess public perception of the severity of the pandemic and its psychosocial impacts, considering age, gender and individual risk factors	Prospective cross-sectional study	969/Estados Unidos	The perception of risk of severe illness from COVID-19 increased with age (*p < * 0.001) and was higher in women (*p < * 0.001).
Hakim et al. (19)	INQUIRY: the Journal of Health Care Organization, Provision, and Financing	To analyze Pakistani healthcare professionals' perceptions of COVID-19-related risks and deaths	Cross-sectional study	462/Paquistão	Physicians had a higher risk perception compared to nurses and paramedics (*p < * 0.05). Female participants (OR 2.01-1.01-4.19) *p < * 0.04 and who tested positive for COVID-19 (OR 2.27-1.22-4.19) *p =* 0.009 were associated with a perceived risk of death from COVID-19.
Alschuler et al. (20)	Multiple Sclerosis and Related Disorders	To understand how people living with multiple sclerosis in the United States experienced distress and perceived risk related to COVID-19 during the first outbreak of the pandemic.	Cross-sectional study	491/Estados Unidos	Greater severity of multiple sclerosis disability was associated with higher perceived risk of dying from COVID-19 (β = 0.15, *p* < 0.01); Having more CDC-defined risk factors for COVID-19 (β = 0.13, *p* < 0.01) was associated with higher perceived risk of dying from COVID-19; Higher anxiety (β = 0.25, *p* < 0.001) and lower positive affect and well-being (β = −0.12, *p* < 0.05) were associated with higher perceived risk of dying from COVID-19.
Kamran et al. (21)	BJPsych Open	To assess people's perception of risks and their adherence to recommended preventive behaviors in relation to COVID-19 infection.	Cross-sectional study	1.861/Irã	Perception of risk in the worsening of COVID-19 disease was higher in the age group over 50 years old [mean 16.8 (SD 1.7) *p < * 0.008] and in married people [mean 16.59 (SD 1.9) *p < * 0.001].
Jamieson et al. (22)	International Journal of Environmental Research and Public Health	Identify associations between ethnic identity and immigration status and perceptions of risk from COVID-19 in the United States.	Cross-sectional study	140.000/Estados Unidos	Identifying as a first- and second-generation Hispanic/Latino individual was associated with an increased perceived risk of dying. Anxiety, discrimination, Spanish language, and age were also associated with higher perceived risk of dying from COVID-19. First- and second-generation Mexicans and other Spanish descendants had higher perceived risk of dying from COVID-19.
Aumala et al. (23)	Frontiers in Public Health	To assess the perceived risk of infection and complications due to COVID-19 in people with hypertension living in a semi-urban city in Ecuador.	Cross-sectional study	260/Equador	In the analysis of the perception of risk of developing complications due to COVID-19, there was no difference between genders.
De Bruin (45)	Journals of Gerontology: Series B: Psychological Sciences and Social Sciences	To examine age differences in COVID-19 risk perceptions, anxiety, and depression.	Cross-sectional study	6.666/Estados Unidos	The older the adults, the higher the perceived risk of dying from COVID-19 (0.18 β= 0.17 *p < * 0.001)
Laires et al. (24)	JMIR Public Health and Surveillance	To examine the association between chronic diseases and severe outcomes after COVID-19 infection, and to explore their influence on people's self-perceptions of risk of worse COVID-19 outcomes.	Cross-sectional study	20.293/Portugal	There was an association between risk perceptions of severe COVID-19 outcomes in participants with cancer (odds ratio [OR] 8.57, 95% CI 5.73–12.81), respiratory diseases (OR 8.25, 95% CI 7.21–9.44) and diabetes (OR 6.17, 95% CI 4.58–8.31). Increasing age, low education level, smoking and worse health status were also associated with the perception of risk of severe COVID-19 disease.
Kohler et al. (25)	Journal of Primary Care and Community Health	To define the impact of the coronavirus pandemic on the behavior and mental health of individuals at high risk of developing a severe course of COVID-19.	Cross-sectional study	12.885/Alemanha	The greater the number of risk diseases, the greater the perception of risk of severity and death from COVID-19. Participants with chronic respiratory, cardiovascular and multiple diseases had greater perceptions of risk of severity and death from COVID-19.
Reynolds et al. (26)	PloS ONE	To measure smokers' trust in COVID-19 information sources and how this trust is associated with perceptions of COVID-19 susceptibility and severity.	Cross-sectional study	2.752/Estados Unidos	People with liberal political orientations had a higher risk perception regarding the severity of COVID-19, as did those who had greater trust in health information and news sources. People aged ≥ 60 years also had a higher risk perception regarding the severity of the disease.
Moniz et al. (27)	Saúde e Pesquisa	Identify factors related to the perception of risk of becoming ill with COVID-19 in adults in the Southeast Region.	Cross-sectional study	2.477/Brasil	The perception of risk of developing the severe form of COVID-19 was higher among females (*p < * 0.001), being in the age group of 18–39 years (*p < * 0.001), having up to elementary education (*p < * 0.001) and having a positive perception of the risk of a family member becoming ill with COVID-19 (*p < * 0.001)
Lolita and Ikhsanudin (28)	Borneo Journal of Pharmacy	To investigate the individual characteristics that influence the perception of COVID-19 risk and efficacy beliefs at different stages of the COVID-19 pandemic	Cross-sectional and analytical	227/Indonésia	The perception of risk of disease severity was greater among entrepreneurs when compared to public sector workers.
Adachi et al. (29)	SSM Population Health	To estimate the association between perceived risk of COVID-19 infection and severe disease and sociodemographic, psychological, and vaccine-related factors, and the level of trust in various sources of information.	Cross-sectional study	29.708/Japão	There was an association between perceived risk of severe disease and increasing age 1.03 1.02–1.03 < 0.001 and people reporting that COVID-19 had a major impact on their lives (OR 1.81; 1.43–2.29), people with underlying diseases 3.17 2.80–3.58 *p* < 0.001.
Al-Raddadi et al. (30)	Journal of Epidemiology and Global Health	To explore the determinants of perceived COVID-19 severity, perceived disease infectivity, and perceived self-efficacy in disease prevention	Cross-sectional study	384/Arábia Saudita	The perceived severity of COVID-19 was lower in males (OR = 0.69, 95% CI = 0.61–0.79; *p* < 0.001) and higher in people who lost relatives to COVID-19 (OR = 2.08, 95% CI = 1.30–3.31; *p* < 0.001)
Luo and Schnall (31)	Social and Personality Psychology Compass	We explored whether perceptions of mortality risk varied by demographic context.	Cross-sectional study	8.339/Estados Unidos	Perceived risk of death from COVID-19 was higher among white women and nonwhite men and women compared to white men. Age and having close relatives who were hospitalized or died from COVID-19 were positively associated with perceived risk of death from COVID-19.
Boonsaeng et al. (32)	Disaster Medicine and Public Health Preparedness	To determine factors associated with perceived risks of infection, as well as perceived risks of hospitalization and death from COVID-19 in Ecuador and Kenya	Cross-sectional study	963/Equador e Quênia	Perceived risk of death from COVID-19 in Kenya was associated with female gender. Perceived risk of hospitalization in Ecuador was associated with male gender and having health insurance. Perceived risk of death from COVID-19 in Ecuador was associated with female gender.
Anson and Eritsyan (33)	International Journal of Adolescence and Youth	To explore the roles of young people's prosocial, self-interested, and controlled motivations to comply with recommended protective behavior during the early stages of the COVID-19 pandemic.	Cross-sectional study	1.376/Rússia	Knowledge of COVID-19 cases on social media, as well as those who required hospitalization, were associated with an increased perception of threat and severity of COVID-19
Kumar and Encinosa (34)	Journal of Racial and Ethnic Health Disparities	To examine racial and ethnic disparities in perceived risks of COVID-19 and in obtaining medical care.	Cross-sectional study	2.135/Estados Unidos	The perception of risk of needing medical care and hospitalization was higher among Black and Latinx people. Asian, Black, and Latinx people had a higher perception of risk of death from COVID-19 when compared to White people.

Among the main factors associated with the perception of risk of severity or death from COVID-19 are: older age—identified by nine studies (17, 18, 21, 22, 24, 26, 29, 32, 45); female sex/gender—identified by six studies (17–19, 27, 30, 32); having previous negative personal or close experiences with COVID-19—identified in four studies (19, 28, 30, 33); having chronic non-communicable diseases (24, 25, 29) or low education level (17, 24, 27)—identified by three studies each; being foreign or of foreign descent (22, 34), being black (17, 34), having worse health status (20, 24) and having anxiety and/or less positive affect and wellbeing (20, 22)—identified in two studies each. Other factors that were mentioned only once were shown in Table 4.

**Table 4:** Summary of the main factors associated with the perception of risk of severity or death from COVID-19, Brazil, 2024.

**Associated factors**	**References**
Older ages	(17, 18, 21, 22, 24, 26, 29, 32, 45)
Sex/gender female	(17–19, 27, 30, 32)
People who have had previous negative personal or family experiences with COVID-19	(19, 28, 30, 33)
Presence of Non-Communicable Chronic Disease	(24, 25, 29)
Low education level	(17, 24, 27)
Foreign people or foreign descendants	(22, 34)
Black people	(17, 34)
Have worse health status	(20, 24)
Having anxiety and/or less positive affect and wellbeing	(20, 22)
Age 18 to 39 years	(26)
Sex/gender male	(32)
Married people	(34)
People with low/middle income	(17)
Doctors	(19)
Having more risk factors for COVID-19	(20)
Smoking	(24)
People with a liberal political orientation	(26)
People who had greater trust in information and news sources	(26)
Have health insurance	(32)

A critical assessment of the methodological quality of the included studies was conducted using the Joanna Briggs Institute (JBI) tool, enabling the verification of rigor and reliability in the analyzed research. The results revealed an overall average methodological quality of 65.8%, with significant variations among studies, whose scores ranged from 25 to 100%. Only one study, by De Bruin and Bennett (17), achieved the maximum score, demonstrating exceptional methodological rigor by meeting all assessed criteria. Other studies exhibiting high methodological quality (≥80%) included those by Shauly et al. (18), Alschuler et al. (20), Kamran et al. (21), and Kohler et al. (25), indicating a more robust evidence base in these investigations. Conversely, studies with lower scores, such as those by Reynolds et al. (26) and Luo and Schnall (31), which obtained only 25%, displayed methodological weaknesses that may potentially compromise the reliability of their findings (Table 5).

**Table 5:** Assessment of the methodological quality of the studies included in the systematic review on the factors associated with the perception of risk of severity or death from COVID-19, according to the databases consulted, Brazil, 2024.

**References**	**Q 1- Were the criteria for inclusion in the sample clearly defined?**	**Q 2- Were the study subjects and the setting described in detail?**	**Q 3- Was the exposure measured in a valid and reliable way?**	**Q 4- Were objective, standard criteria used for measurement of the condition?**	**Q 5- Were confounding factors identified?**	**Q 6- Were strategies to deal with confounding factors stated?**	**Q 7- Were the outcomes measured in a valid and reliable way?**	**Q 8- Was appropriate statistical analysis used?**	**Score (out of 8)**	**Score %**
De Bruin and Bennett (17)	Y	Y	Y	Y	Y	Y	Y	Y	8	100
Shauly et al. (18)	Y	Y	Y	Y	Y	N	Y	Y	7	87.5
Hakim et al. (19)	Y	Y	Y	Y	NEC	NEC	NEC	N	4	50.0
Alschuler et al. (20)	NEC	Y	Y	Y	Y	Y	Y	Y	7	87.5
Kamran et al. (21)	Y	Y	Y	Y	Y	Y	Y	N	7	87.5
Jamieson et al. (22)	Y	Y	Y	Y	Y	N	Y	N	6	75.0
Aumala et al. (23)	Y	NEC	Y	Y	Y	NEC	NEC	N	4	50.0
De Bruin (45)	NEC	Y	Y	NEC	N	N	Y	Y	4	50.0
Laires et al. (24)	Y	Y	NEC	Y	NEC	Y	Y	Y	6	75.0
Kohler et al. (25)	Y	Y	Y	Y	NEC	Y	Y	Y	7	87.5
Reynolds et al. (26)	NEC	NEC	NEC	NEC	NEC	NEC	Y	Y	2	25.0
Moniz et al. (27)	Y	Y	Y	Y	N	N	Y	Y	6	75.0
Lolita and Ikhsanudin (28)	Y	Y	Y	NEC	Y	Y	N	N	5	62.5
Adachi et al. (29)	Y	Y	Y	NEC	NEC	N	Y	Y	5	62.5
Al-Raddadi et al. (30)	Y	NEC	Y	Y	NEC	NEC	Y	Y	5	62.5
Luo and Schnall (31)	NEC	NEC	NEC	NEC	NEC	NEC	Y	Y	2	25.0
Boonsaeng et al. (32)	NEC	NEC	Y	Y	NEC	NEC	Y	Y	4	50.0
Anson and Eritsyan (33)	Y	Y	Y	Y	NEC	NEC	Y	Y	6	75.0
Kumar and Encinosa (34)	NEC	NEC	Y	Y	N	Y	Y	Y	5	62.5
Average score										65.8

Q, Question; Y, Yes; N, No; NEC, Unclear; NA, Not applicable. Source: authors, 2024.

Table 5 shows the results of the methodological quality assessment of the studies included in the review. The studies showed an average score of 65.8%, with the highest score in the studies by De Bruin and Benett (17), Shauly et al. (18); Alschuler et al. (20), Kamran et al. (21), and Kohler et al. (25). The methodological quality of the studies varied significantly, with scores ranging from 25 to 100%.

## 4 Discussion

The aim of this systematic review was to identify factors associated with the perception of risk for severe illness or death from COVID-19 and thus contribute to the formulation of effective strategies to mitigate the impact of future pandemics on the population.

The study evidenced that older individuals exhibit a heightened perception of risk regarding the potential worsening of COVID-19 compared to younger populations. This heightened awareness is likely attributed to the increased vulnerability of older adults to severe forms of the disease, as evidenced by studies highlighting their greater susceptibility to complications (35). In contrast, younger adults, particularly those aged 18 to 39 years, have been found to underestimate the severity of COVID-19, a tendency that may contribute to engaging in riskier behaviors and lower adherence to preventive measures (26). This discrepancy in risk perception across age groups highlights the need for targeted health communication strategies that resonate with the specific concerns and behaviors of different demographic segments.

Moreover, marital status has emerged as another factor influencing risk perception. Findings show that married individuals often report a greater perception of risk (34). This observation could be partially explained by the fact that married individuals are generally older on average than their single counterparts, suggesting that age might play a pivotal role in shaping their heightened awareness. However, it is also possible that the responsibilities and interdependencies associated with marriage contribute to an increased sensitivity to health risks, underscoring the interplay of social and demographic factors in risk assessment.

These findings highlight the complex dynamics between individual characteristics and risk perception, emphasizing the importance of tailoring public health messaging and interventions to address these nuanced differences. By understanding how age, marital status, and other factors influence risk perception, policymakers and health professionals can design more effective strategies to promote adherence to protective measures and reduce the impact of future pandemics.

In the present study, women exhibited a higher perception of the risk of developing severe COVID-19 compared to men. This finding is consistent with the literature, which reveals that women tend to report greater sensitivity to risk perception and fear of life-threatening events compared to men (17). In addition, the results showed that people who identify as African-American, Mexican, of Spanish descent, Hispanic/Latino, as well as first-generation immigrants, reported a significantly higher perceived risk of death due to COVID-19 compared to other population groups (17, 34). This heightened perception of risk can be attributed to a number of factors, including limited access to health services in host countries, especially for those who have migrated recently. The lack of a structured support network in destination countries, coupled with language and cultural barriers, can increase these groups' sense of vulnerability to the complications associated with the disease.

In the context of public health, communication goes beyond the simple transmission of data, promoting an expanded debate on the population's needs, advocating for collective interests, and strengthening the comprehensiveness of care and the intersectoral nature of health actions (36). The WHO emphasizes risk communication and community engagement as essential pillars for successful responses to public health emergencies. Failures in this process can result in loss of public trust, damage to the reputation of institutions, negative economic impacts, and, ultimately, an increase in the number of deaths (37).

In this scenario, it is crucial to adopt tailored health communication strategies for the vulnerable populations, considering their sociocultural particularities and challenges in accessing information (38). Educational campaigns should be developed in multiple languages and disseminated through accessible media for these groups, such as community radios, social media, and printed materials distributed at strategic locations like community centers and health units. In this regard, the involvement of community leaders and healthcare professionals who share cultural experiences with these populations can help spread reliable information and increase adherence to COVID-19 protective measures (39).

In addition, these populations may face difficulties in accessing adequate health information, which contributes to a greater perception of risk and greater apprehension about the impacts of the pandemic. Vulnerable populations, characterized by lower levels of education, income and limited access to information about health risks due to COVID-19 (17, 24, 27). This perception of risk may be related to several factors, including difficulties in accessing reliable health information and a lack of resources to respond effectively to the pandemic. On the other hand, individuals with higher levels of education, particularly those in high-risk occupations, also showed a higher perception of the risk of serious illness caused by COVID-19. Doctors, for example, tend to have a more pronounced perception of this risk due to their professional training, direct exposure to COVID-19 cases and extensive experience in managing critically ill patients. Their in-depth knowledge of the complications associated with the virus strengthens this awareness. However, this heightened perception may be somewhat mitigated by their confidence in their own self-care skills and access to advanced medical resources, which are often not available to more vulnerable groups (19).

Another factor that can impact the perception of risk of developing severe forms of COVID-19 is personal experience with the disease, such as having family members or friends who have died as a result of the infection. This type of experience tends to increase awareness of the fragility of life and the potential for adverse events, intensifying the sense of vulnerability (30). One study revealed that the majority of individuals with chronic illnesses considered themselves to be at high risk of serious complications from COVID-19, with one in four patients with chronic illnesses believing that their condition could worsen due to the virus. This feeling of high risk can be explained by the fact that more than 80% of these individuals have witnessed patients with chronic conditions who, after contracting COVID-19, have progressed to severe stages, resulting in death (23, 40).

In addition to the presence of NCDs, our results indicate that individuals with anxiety have a greater perception of the risk associated with the severity of COVID-19. Together, these findings contribute to the collective understanding of psychological manifestations and their association with the severity of COVID-19, illustrating how mental health status influences individuals' risk perceptions during public health emergencies such as the COVID-19 pandemic. This may occur because a perception of high risk evokes feelings of fear, which can lead individuals to adopt protective measures (44).

The presence of anxiety symptoms in our study is consistent with other studies on psychological distress during the COVID-19 pandemic. Anxiety was found to be more prevalent in populations with a negative perception of COVID-19. This condition was particularly common among older individuals, those with greater intolerance of uncertainty about the disease, less optimistic people and those who experienced greater loneliness. In this sense, health crises such as COVID-19, distress, fear and suffering make the threat seem more immediate and tangible (41). The recognition that fear of the severity and contagiousness of COVID-19 is the most significant stressor associated with the disease in a sample of the general population further reinforces this finding (42).

The liberal rhetoric in some countries often downplayed the severity of the COVID-19 pandemic and opposed containment measures like lockdowns, which contributed to the polarization of information. This, in turn, heightened feelings of insecurity and uncertainty among the public (43). Individuals who expressed greater trust in key sources of information, such as the World Health Organization (WHO), the Food and Drug Administration (FDA), and the media, tended to have higher perceptions of the risk associated with COVID-19. This underscores the importance for healthcare professionals to provide clear guidance on the severity of the disease, as there is a significant ideological divide that undermines trust in conventional information sources (26).

The COVID-19 pandemic was also marked by the widespread dissemination of misinformation, which became a major challenge and further complicated the global health crisis. False or misleading information about the virus, treatments, vaccines, and prevention measures created an environment of fear and uncertainty, which hindered public adherence to health guidelines and, in some cases, worsened the impact of the pandemic. Misinformation also had economic consequences, eroding trust in health institutions and science, which ultimately influenced health insurance costs and medical agreements (7). The uncertainty surrounding the disease placed additional pressure on healthcare systems, with many individuals seeking ineffective treatments or delaying necessary care. This led to an increased demand for medical services and a subsequent rise in costs.

The methodological quality of the reviewed studies exhibited remarkable diversity, reflecting a wide spectrum of research rigor and adherence to best practices. Scores ranged dramatically from 25 to 100%, highlighting the stark contrast between studies that exemplified methodological excellence and those that fell short of established quality standards. This variation underscores the importance of critical evaluation when interpreting research findings.

At the pinnacle of methodological rigor, the study by De Bruin and Bennett (17) stood out, achieving a perfect score of 100% and setting a gold standard for research quality. Close behind, studies by Shauly et al. (18), Alschuler et al. (20), Kamran et al. (21), and Kohler et al. (25) also demonstrated high methodological quality, with scores of 80% or above. These high-scoring studies provide a more reliable evidence base, offering greater confidence in their findings and conclusions. In contrast, studies at the lower end of the spectrum raise significant concerns about the reliability of their results. These methodological weaknesses not only compromise the internal validity of the affected studies but also limit the generalizability of their findings and their applications in healthcare.

The clarity and consistency of research findings are crucial for accurate interpretation. However, studies in our review exhibited concerning discrepancies between their tabulated results and textual descriptions (19, 22, 28). The conclusions drawn from the data presented in tables often diverged from the interpretations offered in the main text, which might difficult the reader in the reading and raise questions about the overall reliability of the studies' findings. While the results in the table suggest a certain conclusion, the text appears to indicate a different direction, creating a lack of coherence and clarity in the information provided. This incongruity can lead to misinterpretations and hinder readers' understanding of the true findings of the research.

One of the main limitations of this study lies in the methodological heterogeneity of the included studies, which utilize different theoretical models to investigate the perceived risk of severe illness or death from COVID-19. This diversity in theoretical approaches may have hindered direct comparisons of results and the formulation of more robust and generalizable conclusions. These factors, combined with the lack of standardization in definitions and measures of risk perception, may have introduced bias into the findings, complicating the precise identification of factors associated with COVID-19 risk perception.

Finally, it is important to consider that the review was limited to studies available up to the time of the research, which may introduce a temporal bias in the interpretation of results, given the dynamic nature of the pandemic and the ongoing evolution of scientific knowledge about COVID-19. Additionally, several studies were conducted using online questionnaires filled out independently by participants, which raises concerns such as the potential for subjective self-assessment when professional interviewer supervision is absent. Individuals with limited internet access were likely not included in the study, creating a selection bias in the studied population. Another concern is the overrepresentation of women in most studies. The selection bias and the overrepresentation of specific groups suggest that the majority of studies may not be representative of the actual population.

The findings from this study not only enhance our understanding of COVID-19 risk perception but also open up possibilities for exploring this phenomenon in other contexts. This knowledge can improve preparedness and response from local health systems to new pandemics and help address current health problems and diseases. The influence of sociodemographic, emotional, and cultural factors on risk perception, as discussed throughout this study, can be applied to various areas to enhance interventions that promote preventive behaviors and adherence to safety measures.

Furthermore, it is crucial for future research to incorporate more standardized methodological approaches and precise measures to capture the diverse influences that shape risk perception. This approach will lead to more robust and comparable results across different studies and contexts, ultimately contributing to more effective risk communication and management strategies in public health. By building on these findings and refining research methodologies, it is also necessary to develop a more comprehensive understanding of risk perception through strategic research approaches such as community-based participatory research. This is an equitable study approach where researchers, organizations, and community members collaborate on all aspects of a research project.

Such collaboration is essential for understanding all determinants that shape risk perception. This inclusive method ensures that diverse perspectives are incorporated, leading to a more comprehensive and nuanced understanding of how individuals and communities perceive and respond to risks. By engaging community members as active participants in the research process, it is gained deeper insights into the cultural, social, and contextual factors that influence risk perception, ultimately leading to more effective and tailored public health strategies.

## 5 Conclusion

The study revealed that during the COVID-19 pandemic, several factors influenced the perception of risk for severe illness or death from COVID-19. These factors included advanced age, female gender, personal experiences or witnessing adverse COVID-19 outcomes, the presence of chronic non-communicable diseases, and lower educational attainment. The findings indicate a concerning situation, as individuals with these characteristics may have overestimated the infectiousness of the virus. This heightened perception likely played a vital role in motivating the adoption of health-protective behaviors.

## Data Availability

The original contributions presented in the study are included in the article/supplementary material. Further inquiries can be directed to the corresponding authors.
